# Factors influencing body weight one year after bariatric surgery

**DOI:** 10.1097/MD.0000000000033111

**Published:** 2023-03-17

**Authors:** Afnan Sameer Azhri, Asma Almuqati, Firas Azzeh, Nuha Alamro, Wedad Azhar, Alaa Qadhi, Khloud Ghafouri

**Affiliations:** a Department of Clinical Nutrition, King Abdullah Medical City, Makkah, Saudi Arabia; b Department of Clinical Nutrition, Faculty of Applied Medical Sciences, Umm Al-Qura University, Makkah, Saudi Arabia.

**Keywords:** bariatric surgery, cholecystectomy, obesity, weight loss

## Abstract

The significant outcome of bariatric surgery (BS) is weight loss, which may be affected by many factors, such as initial body weight before surgery, sex, and dietary intake. Moreover, rapid weight loss is associated with an increased incidence of postsurgical cholelithiasis. To investigate the observed weight loss outcomes during the first year after BS, we investigated the factors that may influence weight loss and to detect the efficacy of prophylactic ursodeoxycholic acid against gallstone formation. This was a retrospective cohort study of all patients with morbid obesity who underwent BS in the hospital and completed a 1-year follow up. Patients with a previous BS or a history of cholecystectomy before BS were excluded. Data were extracted from the medical records at multiple postoperative intervals. There was significant weight loss in terms of percentage of excess weight loss and reduction in body mass index postoperative. A significant correlation was found between the percent of excess weight loss and age, initial body mass index, and initial weight, but there was no significant correlation with sex or type of surgery. The incidence of postoperative cholecystectomy is almost negligible. A significant association was found between age and weight loss after BS. ursodeoxycholic acid is an effective prophylaxis to decrease the incidence of cholecystectomy after BS.

## 1. Introduction

Obesity has become a significant health challenge worldwide and places an increased burden on the healthcare system. The prevalence of obesity in Saudi Arabia is approximately 34.8% and 35.6% in women.^[1]^ Weight reduction has been recognized as a critical factor in the prevention, management, and mitigation of the adverse effects of many comorbidities such as type 2 diabetes, hypertension, cardiac disease, dyslipidemia, and sleep apnea. Furthermore, weight loss also decreases morbidity and mortality in obese patients with obesity.^[2]^

Weight reduction in obese individuals can be achieved using surgical or nonsurgical approaches. Nonsurgical treatment is a multicomponent approach involving diet restriction, lifestyle modification, and exercise, as well as pharmacological and behavioral therapy. nonsurgical treatment usually induces short-term weight loss, but the resulting weight loss is rarely permanent because of poor compliance.^[3]^

Bariatric surgery (BS) is an effective and long-lasting treatment for patients with obesity who cannot maintain healthy body weight.^[4]^ BS reduces the risk of mortality by 51%,^[5]^ and weight loss surgery can help address many obesity related diseases, including high blood pressure, dyslipidemia, type 2 diabetes, certain cancers, heart disease, joint problems, and sleep apnea.^[6]^

In 2019, 833,687 bariatric surgeries were performed globally. However, due to safety and effectiveness issues, these surgeries can result in short- and long-term complications.^[7]^

Weight loss is the major outcome of BS, but it can be affected by many factors including sociodemographic, behavioral problems, genetics, patient status, and the surgical technique itself.^[8]^ However, few data are available on the factors affecting weight loss or weight stability after BS.

Nevertheless, there are potential nutritional complications, including dumping syndrome with reactive hypoglycemia; protein malnutrition; nutritional and mineral deficiencies such as vitamins A, C, D, E, B, and K; folate, iron, magnesium, thiamin, and zinc; and regaining weight.^[9]^ Patients develop these complications because of rapid changes in weight, malabsorption, or low dietary intake.^[10]^ Furthermore, recent studies have found cholelithiasis after various forms of BS, with a worldwide prevalence rate between 2% and 50% worldwide.^[11]^ A recent study carried out in Saudi Arabia found a 3.5% prevalence of cholelithiasis after BS.^[11]^ Cholelithiasis is also associated with rapid weight loss.^[12]^

BS may result in multiple physiological factors that influence gallstone formation. Hyper saturation of bile with cholesterol increases mucin production, gallbladder hypomotility contributes to gallstone formation, and division of the hepatic branch of the vagus nerve affects the gallstone as well.^[13]^ The management of cholelithiasis in bariatric patients remains unspecified and several therapeutic strategies have been used. Furthermore, there is no protocol for performing cholecystectomy at the time of BS.^[14]^ Ursodeoxycholic acid (UDCA) has been suggested as a prophylaxis for reducing gallstone formation.^[15]^ However, it is unclear whether this treatment is effective in Saudi Arabian patients.

Therefore, this study aimed to investigate the factors influencing weight change during the year after BS and to detect the efficacy of prophylactic UDCA in preventing gallstone formation related to rapid weight loss among BS patients.

## 2. Methods

For this retrospective cohort study of patients with morbid obesity who underwent BS in our hospital, 162 participants were recruited. The sample size was calculated based on a statistical power of 80%, a confidence level of 95%, and a margin of error of 5%. These participants were aged 18 to 65 years, and they completed 1 year of follow up at the nutrition clinic between May 2018 and May 2020. The exclusion criteria were BS outside the timeframe of the study, age > 65 years or < 18 years, cholelithiasis before surgery, and cholecystectomy before BS.

Data were extracted from the electronic medical records of the patients who met the inclusion criteria. These patients were followed from the day of surgery until the end of the first year. Patients were examined immediately after surgery (to determine baseline) and at intervals of 2 weeks and 3, 6, 9, and 12 months after surgery. The data included age, sex, type of surgery, initial weight, weight at follow up, height, and body mass index (BMI), calculated as weight in kilograms divided by the square of height in meters (CDC, 2020). The ideal body weight was calculated based on the upper limit of normal BMI (i.e., 24.9 kg/m^2^) multiplied by the square of height in meters.^[16]^ The percentage of excess weight loss (%EWL) was calculated at 6, 9, and 12 months postoperatively by dividing the difference between the initial and final weights by the difference between the initial and ideal body weights multiplied by 100.^[17]^ Successful weight loss was calculated based on a 50% EWL or >.^[18]^

The postoperative occurrence of cholecystectomy was recorded in patients who developed gallstones during the first year after BS.

Statistical analysis was performed using Statistical Package for the Social Sciences (IBM SPSS Statistics for Windows, Version 23.0. Armonk, NY: IBM Corp). Categorical variables were displayed as frequencies and percentages. The minimum, maximum, mean, and standard deviation were used to present continuous variables. Paired *t* tests were used to identify significant differences in the findings preoperatively and at 6, 9, and 12 months postoperatively. Pearson correlation coefficient was used to test for associations between the continuous variables. The chi-square test was used to check for significant associations between categorical variables. Cohen *d* was used to determine the magnitude of the surgery effect and was calculated by subtracting the mean of 1 group from the other (*M*1–*M*2) and dividing the result by the pooled standard deviation (SD). A small effect size is equal to (d = 0.2), medium effect size is equal to (d = 0.5), and large effect size is equal to (d ≥ 0.8).^[19]^ The level of significance was set at *P* ≤ .05. The protocol of this study was approved by the hospital institutional review board, number 21 to 757.

## 3. Results

A total of 162 participants were included in this study. Table 1 displays the baseline characteristics of the participants and the surgery types that they had undergone.

**Table 1:** Baseline characteristics of the participants (n = 162).

Baseline characteristic	n	%
Sex		
Male	52	32.1
Female	110	67.9
Type of surgery		
Laparoscopic sleeve gastrectomy (LSG)	139	85.8
Roux-en-Y gastric bypass (RYGB)	9	5.6
Mini-gastric bypass (MGB)	14	8.6
Age (yr)		
Mean ± SD	40.6 ± 10.4	
Minimum	15.00	
Maximum	63.00	
Baseline BMI		
Mean ± SD	49.06 + 7.39	
Baseline weight (kg9		
Mean ± SD	129.1 ± 24.3	

BMI = body mass index, LSG = laparoscopic sleeve gastrectomy, MGB = mini gastric bypass, RYGB = Roux-en-Y gastric bypass.

**Figure 1.**
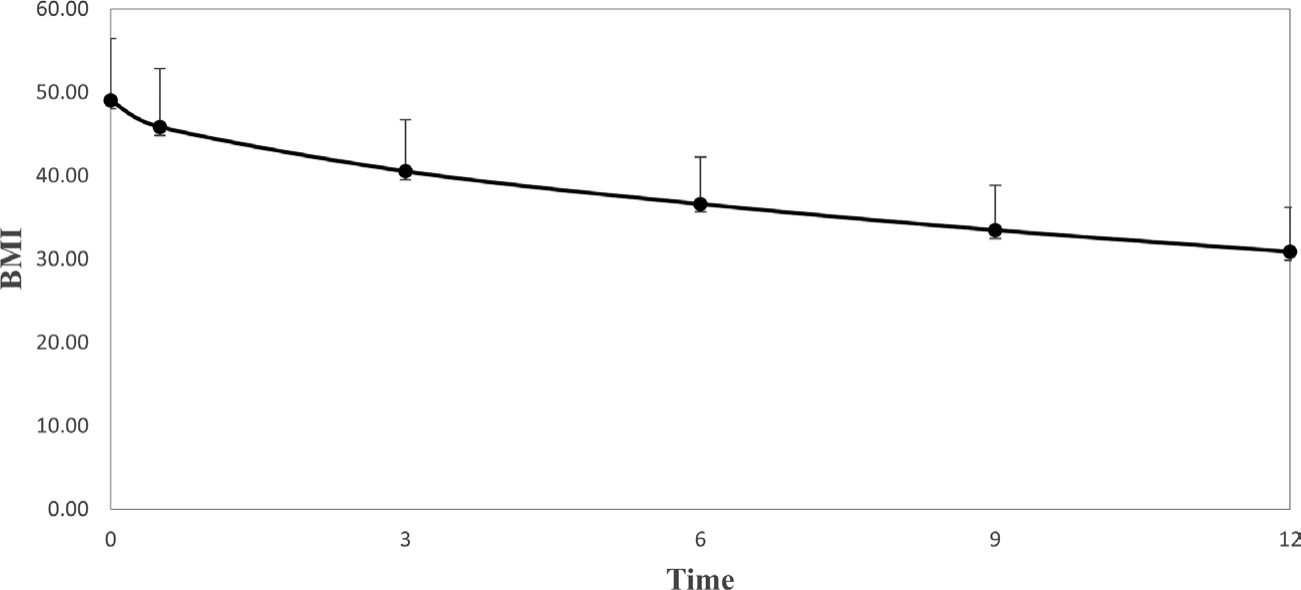
BMI Trend over 1 year post-op. BMI = body mass index.

Figure 1 shows the trend in BMI pre- and postoperative over 1 year. A decrease in BMI was observed over 1 year. The mean and standard deviation of the BMI before surgery were 49.1 ± 7.4. Two weeks after surgery they were 45.9 ± 7; at 3 months they were 40.6 ± 6.2; at 6 months they were 36.6 ± 5.7; at 9 months, 33.5 ± 5.4; and at 12 months, 30.9 ± 5.3.

**Figure 2.**
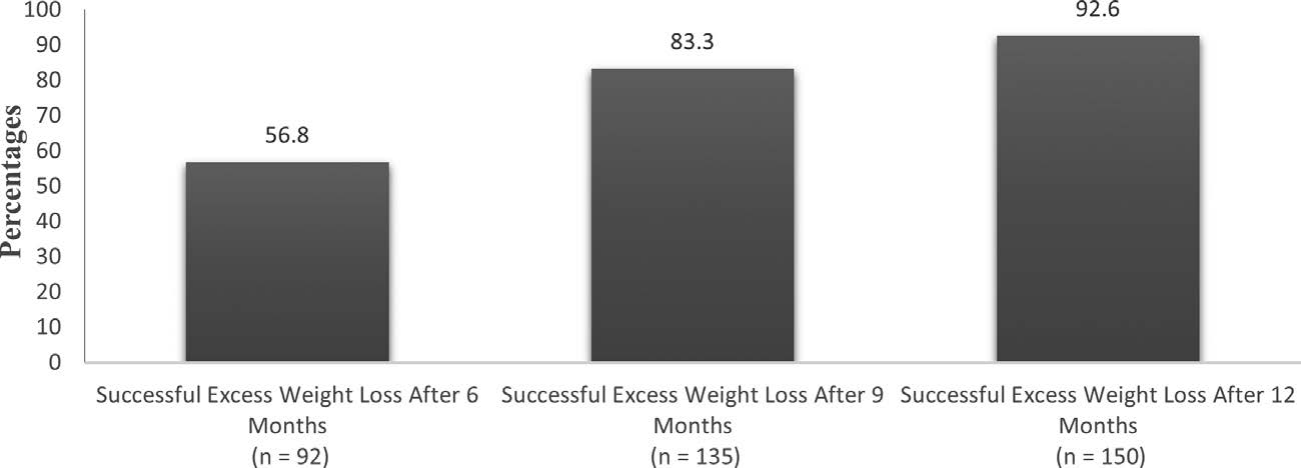
Successful excess weight loss over 1 year.

Figure 2 demonstrates the rate of successful loss of excess weight (defined as loss of 50% or more of excess weight) among participants over 1 year. More than half (56.8%) had successful excess weight loss 6 months postoperatively, whereas 70 (43.2%) failed. At 9 months postoperatively, 135 (83.3%) patients had successful excess weight loss, whereas 27 (16.7%) failed. At 12 months postoperatively, 150 (92.6%) patients had successful excess weight loss, whereas 12 (7.4%) had failed.

Table 2 lists %EWL overtime. The mean and standard deviation for %EWL at 6 months, 9 months, and 12 months postoperatively were 52.8 ± 14.1, 66 ± 15.7, and 76.9 ± 17.5, respectively. There was a significant difference in the mean %EWL at 6 months compared with that at 9 months (*P* < .001) and 12 months (*P* < .001). There was also a significant difference in the mean %EWL at 9 months compared with that at 12 months (*P* < .001). Cohen *d* effect sizes were calculated for each pair for comparison. There was a large effect size (0.89) when comparing the EWL at 6 months to the EWL at 9 months, and there was a large effect size (1.52) when comparing the EWL at 6 months to the EWL at 12 months postoperatively. There was a medium effect size (0.65) when comparing the EWL at 9 months to the EWL at 12 months postoperatively.

**Table 2:** Comparison of excess weight loss over time.

pair compared	Mean ± SD	*P* value	Cohen d effect size
Percentage of excess weight loss 6 mo post-op	52.8 ± 14.1	<.001*	0.89
Percentage of excess weight loss 9 mo post-op	66 ± 15.7		
Percentage of excess weight loss 12 mo post-op	76.9 ± 17.5	<.001*	1.52
Percentage of excess weight loss 9 mo post-op	66 ± 15.7	<.001**	0.65
Percentage of excess weight loss 12 mo post-op	76.9 ± 17.5		

*P* values were determined by paired *t* test.

* Significant difference *P* < .001 compared with 6 months postoperatively.

** Significant *P* < .001 when compared with 9 months post-op.

Table 3 shows factors associated with excess weight loss across intervals. No significant association was found between excess weight loss and either sex or type of surgery across any interval. Age displayed a weak significant negative correlation with EWL only at 6 months post-op (correlation coefficient = −0.18, *P* = .024). A significant weak negative correlation was found between initial weight and EWL at both 9 months (correlation coefficient = −0.169, *P* = .031) and 12 months (correlation coefficient = −0.199, *P* = .011). A significant medium negative correlation was found between initial BMI and EWL in all time intervals: 6 months (correlation coefficient = −0.31, *P* < .001), 9 months (correlation coefficient = −0.35, *P* < .001), and 12 months (correlation coefficient = −0.33, *P* < .001).

**Table 3:** Factors associated with excess weight loss.

Factor	Time interval for measuring excess weight loss percentage					
	6 Months post-op	P value	9 Months post-op	P value	12 Months post-op	P value
Sex		.156		.08		.253
Male	55.1 ± 14.9		69.2 ± 14.1		79.2 ± 13.9	
Female	51.7 ± 13.7		64.6 ± 16.2		75.8 ± 18.9	
Type of surgery		.276		.175		.674
Laparoscopic sleeve gastrectomy	53.5 ± 13.6		66.4 ± 15.1		77.1 ± 16.9	
Roux-en-Y gastric bypass	46.7 ± 17.3		57 ± 20.3		71.9 ± 20.3	
Mini-gastric bypass	49.9 ± 17.2		68.8 ± 17.4		77.9 ± 22	
Factor	Correlation coefficient	*P* value	Correlation coefficient	*P* value	Correlation coefficient	*P* value
Age	−0.18	.024*	−0.92	.244	−0.107	.173
Initial Weight	−0.148	.059	−0.169	.031*	−0.199	.011*
Initial BMI	−0.31	<.001*	−0.35	<.001*	−0.33	<.001*

*P* values were determined by paired *t* test.

BMI = body mass index.

* Significant difference *P* < .001 compared with 6 months postoperatively.

** Significant *P* < .001 when compared with 9 months post-op.

**Figure 3.**
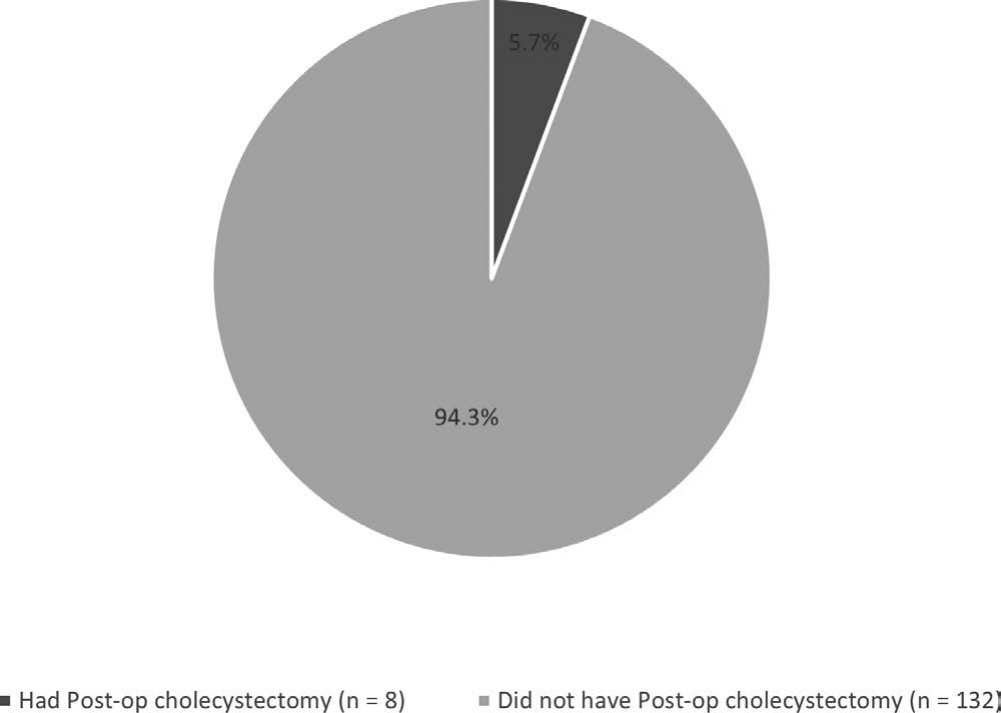
Prevalence of post-op cholcystectomy.

Twenty-two (13.6%) participants underwent cholecystectomy before BS, whereas 140 (86.4%) did not. Among the 140 patients, eight (5.7%) underwent cholecystectomy after BS (Fig. 3).

## 4. Discussion

The goal of the present study was to observe weight loss outcomes during the first year after BS, investigate the factors that may influence weight loss, and measure the incidence of cholecystectomy post-BS.

The results showed that the patients’ BMIs dropped from pre-op to the postoperative baseline and at intervals of 2 weeks and 3, 6, 9, and 12 months. As a result, the mean BMI declined from 49.06 ± 7.39 to 30.91 ± 5.32, causing a change of rating from “morbidly obese” to “overweight.” Similarly, other reports have found a clear downward trend in BMI post-BS.^[20]^

Our results showed successful weight loss (defined as ≥ 50% EWL) among participants over 1 year. At 6 months postoperatively, 56.8% of the participants had successful EWL, and at 9 months, 83.3% had successful EWL. By 12 months postoperatively, 92.6% of the patients had successful EWL. This result aligns well with previous studies, wherein 93% of patients experienced ≥ 50% EWL in 1 to 2 years.^[18]^ Another study found that the mean %EWL was approximately 60% 1 year after surgery.^[21]^

This study found a positive correlation between excess weight loss and time, expressed as %EWL. Furthermore, the study observed that participants’ weight loss was continuous during the first year. There was a significant difference in the mean %EWL at 6, 9, and 12 months postoperatively (*P* < .001). The effect size of weight loss over time was large, and this is clinically meaningful given that weight loss is linked to improvements in overall health. By observing the results of this study within 3 parameters – BMI, %EWL, and weight – there was no noticeable weight stability or weight plateau; the metrics showed a continuous decline throughout the year. This is explained by the compulsory effect of the surgery and the patients’ commitment to postoperative nutritional instructions and exercise, as well as the increase in their morale and enthusiasm due to weight loss.

Regarding the factors that may influence weight loss during the first year after surgery, limited data are available to compare the outcomes of gastric bypass and laparoscopic sleeve gastrectomy (LSG). We found that the type of operation – LSG, mini gastric bypass, or Roux-en-Y gastric bypass (RYGB) – had no significant effect on weight loss in any follow up period within 1 year of the operation. Other studies have shown that the mean %EWL 1 year after surgery was significantly lower after LSG than after laparoscopic Roux-en-Y gastric bypass (49% vs 61%)^[22,23]^found that the %EWL for one anastomosis gastric bypass (OAGB) versus LSG was 66.87 ± 10.87 versus 63.97 ± 13.24, respectively, after 1 year,^[24]^ while patients who underwent RYGB had a greater %EWL after 6, 12, and 24 months compared to patients who underwent LSG. In our findings, this is attributable to the small sample size and the predominance of lap-sleeve gastrectomy among the subjects.

This study also found no significant sex disparity in %EWL. Overall, these findings are in accordance with^[25]^ this study reported that there was no significant influence of sex on the percentage of total weight loss, percentage of body mass index loss, and percentage of excess body mass index loss (%EBMIL) at 12, 24, and 36 months after surgery. There was also no significant difference between males and females in the mean %EBMIL at 24 months.^[26]^ Our results show that there was a weak statistically significant negative correlation between age and %EWL only 6 months after surgery. Similarly, it has been found that no significant association between %EWL and age at 6, 12, or 24 months of follow up.^[24]^, however, the opposite has been reported by, identifying a statistically significant difference in %EBMIL 12 months after surgery between both groups; the patients younger than 45 years old with higher BMIs lost more weight than older patients, possibly because of the higher basal metabolic rate among younger patients.^[27]^ However, in our study, the mean age of the participants was 40 ± 10 years, with little variation in age.

To test the effect of initial BMI on postoperative weight loss in terms of %EWL, this study found a significant negative correlation, which means that patients with higher BMIs before surgery fared poorly after BS. However, it is essential to keep in mind that the greater an individual’s obesity, the greater the total – and thus, excess – body weight. Individuals with higher initial BMIs are less likely to achieve high %EWL^[28]^; this finding has been corroborated by several studies with similar results. It has been reported that individuals with higher initial BMIs showed greater total body weight loss but a lower %EWL at all follow up intervals.^[28]^ Another study found that increased initial BMI was associated with increased percentage of total weight loss and decreased %EWL 2 years after surgery.^[18]^ This means that individuals with higher BMIs before surgery needed more time to achieve successful %EWL than those with lower BMIs.

There are multiple ways to report weight loss outcomes after BS, none of which have been recognized as a standard approach.^[29]^ In this study, we used the %EWL, which is the most commonly used method for measuring and reporting weight loss outcomes in the bariatric surgery literature.^[29]^ Most previous studies have measured the effect of preoperative BMI on %EWL after BS, but there is little evidence available on the effect of baseline weight on %EWL after surgery.

Despite the significant expansion in the rates of bariatric surgery and the advantages after the operation, it has been reported in many recent and scientifically proven studies, several complications may occur after bariatric surgery, including early, and late complications. And its severity varies according to the type of surgery, whether LSG, OAGB, RYGB, gastric balloons, or biliopancreatic diversion with duodenal switch.^[30]^

Based on recent meta-analysis, the frequency of hemorrhage, risk of gastric ulceration, and intraabdominal abscess formation was higher with RYGB than with LSG. In addition, RYGB had a higher rate of internal hernia and risk of anastomotic leaks than that observed in LSG. On the other hand, the incidence of gastroesophageal reflux disease after LSG was greater than that in RYGB.^[31]^ Also, a study confirmed that increase risk of gastroesophageal reflux disease and Barrett esophagus post LSG.^[32]^ The rate of reoperation was higher in the patient who underwent RYGB as compared to those who had LSG. Also, the risk of other complications such as gastric outlet obstruction wound infection, pneumonia, dehydration, and stricture formation was observed more in RYGB.^[31]^

In addition, a study showed that dumping syndrome occurs with any gastric procedure but more expected after RYGB.^[10]^ Nutritional deficiencies are less common with LSG compared with malabsorptive surgeries.^[10]^ A recent study proved that standardization of biliopancreatic limb length in OAGB 150 to 180 cm is effective in term of comorbidity improvement, %EWL and low malnutrition after surgery.^[33]^

Several studies have shown that gallstones may develop because of rapid weight loss after BS.^[11,12,15,34]^ A recent randomized control trial (RCT) showed that the malabsorptive procedure had a higher rate of cholelithiasis than restrictive surgeries^.[35]^

The underlying mechanism is not fully understood; however, a pathogenic mechanism has been proposed. Rapid weight loss changes the metabolism of cholesterol, such that cholesterol is mobilized from adipose tissues and excreted in the bile. Subsequently, the cholesterol level in the bile is so high that bile salts cannot dissolve it.^[15]^ In addition to hypomotility of the gall bladder, increased prostaglandins and arachidonic acid, as well as increased secretion of calcium and mucin, promote the formation of cholesterol crystals.^[12]^ To date, 2 approaches have been used to manage gallstones in bariatric patients: perioperative cholecystectomy and UDCA. However, perioperative cholecystectomy is no longer recommended because it is associated with longer hospital stays, higher complication rates, and longer operating times.^[12]^ One surprising finding in our study is that only 8 (5.7%) of the subjects developed gallstones after BS, even though 150 (92.6%) of them achieved successful EWL during the first year. This is because our center used UDCA as a prophylactic agent against cholelithiasis; 300 mg UDCA was administered twice daily from week 2 until 6 months postoperatively. This is consistent with the results of,^[15]^ who found a significant decrease in the incidence of cholelithiasis after BS from 22% in the placebo group to 6.5% in the group treated with UDCA. The same study found that a higher incidence of gallstones after weight loss surgery occurred during the peak weight loss phase. This encouraged researchers to use UDCA as prophylaxis in the first 6 months after surgery. Moreover, the study found that 6 months after surgery, the treatment group had a significantly lower incidence of gallstones than the control group (6.8% vs 22.2%, respectively; *P* = .028). This pattern was maintained until the 12th month (5.8% vs 14.7%, respectively; *P* = .031).^[31]^ In addition, this study showed that at the 12-month postoperative follow-up, there was a significantly decreasing rate of gallstone formation in the UDCA group compared with control group 4 (4.2%) and 24 patients (25.2%), respectively. And that proved the regular intake of UDCA 6 months after surgery reduced incidence of cholelithiasis after OAGB.^[35]^ UDCA, a hydrophilic secondary bile acid, increases biliary acids that help to form biliary micelles and vesicles, which decreases bile lithogenicity, biliary secretion of cholesterol and mucin, cholesterol saturation, and intestinal absorption.^[36]^ Our study corroborates the findings of previous studies that indicated the effectiveness of UDCA in lowering the incidence of cholelithiasis post-BS.

The limitations of the current study were that most of the participants were female, the predominant surgery was LSG, participants’ diet history and physical activity were not collected, and how they affected weight loss was not studied. Additionally, there were no recorded data on patients’ adherence to UDCA use. One of the strengths of this study is that it is the first in Saudi Arabia to measure the factors that play a significant role in weight loss after BS. Moreover, the study was conducted in a specialized surgery center in King Abdullah Medical City, which is one of the largest medical centers in the kingdom and a pioneer in BS. It must also be acknowledged that the study was conducted during the COVID-19 pandemic, when resources were limited in terms of sample availability, required data, and patients’ commitment to follow-up visits after their operations.

Future research should seek to achieve a closer balance between the number of both sexes in the sample and among the types of operations that will be studied, such that their effect on weight loss will be meaningful. The effects of physical activity, adherence to a healthy diet, smoking, emotional state, and ethnicity should be explored in future studies. Furthermore, as noted above, future research should measure participant adherence to the UDCA regimen and its effect on the formation of post-BS gallstones at individual levels.

## 5. Conclusion

Weight loss after BS is affected by many variables that were measured at multiple intervals during the first year after surgery. Sex and type of surgery did not affect weight loss, but there was a difference based on age. In addition, there was a significant negative association between initial weight and initial BMI and %EWL. The current study also confirms the effectiveness of UDCA in decreasing the incidence of cholecystectomy after BS.

## Acknowledgments

The authors would like to thank the Deanship of Scientific Research at Umm Al-Qura University for supporting this work by Grant Code (22UQU4310486DSR01).

## Author contributions

**Conceptualization:** Afnan Sameer Azhri.

**Data curation:** Asma Almuqati, Nuha Alamro.

**Formal analysis:** Firs Azzeh, Wedad Azhar.

**Investigation:** Asma Almuqati, Nuha Alamro.

**Methodology:** Asma Almuqati, Nuha Alamro.

**Supervision:** Alaa Qadhi, Khloud Ghafouri.

**Validation:** Alaa Qadhi.

**Visualization:** Firs Azzeh, Wedad Azhar.

**Writing – original draft:** Afnan Sameer Azhri.

**Writing – review & editing:** Khloud Ghafouri.
